# Respiratory Support Practices for Bronchiolitis in the Pediatric Intensive Care Unit

**DOI:** 10.1001/jamanetworkopen.2024.10746

**Published:** 2024-05-10

**Authors:** Jonathan H. Pelletier, Danielle E, Maholtz, Claire M. Hanson, Ryan A. Nofziger, Michael L. Forbes, James B. Besunder, Christopher M. Horvat, Christopher K. Page-Goertz

**Affiliations:** 1 Division of Critical Care Medicine, Department of Pediatrics, Akron Children’s Hospital, Akron, Ohio; 2 Department of Pediatrics, Northeast Ohio Medical University College of Medicine, Rootstown, Ohio; 3 Rebecca D. Considine Research Institute, Akron Children’s Hospital, Akron, Ohio; 4 Division of Pediatric Critical Care Medicine, Department of Critical Care Medicine, UPMC Children’s Hospital of Pittsburgh, Pittsburgh, Pennsylvania

## Abstract

**Importance:**

Admissions to the pediatric intensive care unit (PICU) due to bronchiolitis are increasing. Whether this increase is associated with changes in noninvasive respiratory support practices is unknown.

**Objective:**

To assess whether the number of PICU admissions for bronchiolitis between 2013 and 2022 was associated with changes in the use of high-flow nasal cannula (HFNC), noninvasive ventilation (NIV), and invasive mechanical ventilation (IMV) and to identify factors associated with HFNC and NIV success and failure.

**Design, Setting, and Participants:**

This cross-sectional study examined encounter data from the Virtual Pediatric Systems database on annual PICU admissions for bronchiolitis and ventilation practices among patients aged younger than 2 years admitted to 27 PICUs between January 1, 2013, and December 31, 2022. Use of HFNC and NIV was defined as successful if patients were weaned to less invasive support (room air or low-flow nasal cannula for HFNC; room air, low-flow nasal cannula, or HFNC for NIV).

**Main Outcomes and Measures:**

The main outcome was the number of PICU admissions for bronchiolitis requiring the use of HFNC, NIV, or IMV. Linear regression was used to analyze the association between admission year and absolute numbers of encounters stratified by the maximum level of respiratory support required. Multivariable logistic regression was used to analyze factors associated with HFNC and NIV success and failure (defined as not meeting the criteria for success).

**Results:**

Included in the analysis were 33 816 encounters for patients with bronchiolitis (20 186 males [59.7%]; 1910 patients [5.6%] aged ≤28 days and 31 906 patients [94.4%] aged 29 days to <2 years) treated at 27 PICUs from 2013 to 2022. A total of 7615 of 15 518 patients (49.1%) had respiratory syncytial virus infection and 1522 of 33 816 (4.5%) had preexisting cardiac disease. Admissions to the PICU increased by 350 (95% CI, 170-531) encounters annually. When data were grouped by the maximum level of respiratory support required, HFNC use increased by 242 (95% CI, 139-345) encounters per year and NIV use increased by 126 (95% CI, 64-189) encounters per year. The use of IMV did not significantly change (10 [95% CI, −11 to 31] encounters per year). In all, 22 381 patients (81.8%) were successfully weaned from HFNC to low-flow oxygen therapy or room air, 431 (1.6%) were restarted on HFNC, 3057 (11.2%) were escalated to NIV, and 1476 (5.4%) were escalated to IMV or extracorporeal membrane oxygenation (ECMO). Successful use of HFNC increased from 820 of 1027 encounters (79.8%) in 2013 to 3693 of 4399 encounters (84.0%) in 2022 (*P* = .002). In all, 8476 patients (81.5%) were successfully weaned from NIV, 787 (7.6%) were restarted on NIV, and 1135 (10.9%) were escalated to IMV or ECMO. Success with NIV increased from 224 of 306 encounters (73.2%) in 2013 to 1335 of 1589 encounters (84.0%) in 2022 (*P* < .001). In multivariable logistic regression, lower weight, higher Pediatric Risk of Mortality III score, cardiac disease, and PICU admission from outside the emergency department were associated with greater odds of HFNC and NIV failure.

**Conclusions and Relevance:**

Findings of this cross-sectional study of patients aged younger than 2 years admitted for bronchiolitis suggest there was a 3-fold increase in PICU admissions between 2013 and 2022 associated with a 4.8-fold increase in HFNC use and a 5.8-fold increase in NIV use. Further research is needed to standardize approaches to HFNC and NIV support in bronchiolitis to reduce resource strain.

## Key Points

**Question:**

Are changes in high-flow nasal cannula (HFNC) and noninvasive ventilation (NIV) use associated with an increase in pediatric intensive care unit (PICU) admissions for bronchiolitis?

**Findings:**

In this cross-sectional study of 33 816 encounters for patients aged younger than 2 years with bronchiolitis across 27 PICUs between 2013 and 2022, PICU admissions increased by 350 per year. This was associated with a simultaneous increase of 242 HFNC and 126 NIV admissions per year, but no significant change in invasive mechanical ventilation admissions.

**Meaning:**

Findings of this study suggest that increased use of HFNC and NIV is associated with increased PICU admissions for bronchiolitis.

## Teaser

This cross-sectional study examines whether an increase in pediatric intensive care unit admissions is associated with changes in the use of high-flow nasal cannula and noninvasive ventilation in children with bronchiolitis aged younger than 2 years.

## Introduction

Bronchiolitis is a viral lower respiratory tract infection (LRTI), commonly caused by respiratory syncytial virus (RSV).^1,2,3^ Bronchiolitis accounts for over 2 million outpatient visits in the US annually.^4,5^ It is the most common cause of hospitalizations in US children aged younger than 2 years.^6,7^ The viruses that cause bronchiolitis are transmitted more effectively in dry air and indoor settings, leading to annual winter epidemics.^5,6,7,8,9,10,11,12,13^ High case burden combined with strong seasonality has recently resulted in pediatric bed shortages, particularly among pediatric intensive care units (PICUs).^14,15,16^

Whether changes in noninvasive ventilation (NIV) and high-flow nasal cannula (HFNC) therapy use are contributing to PICU bed shortages is an area of debate.^17,18^ Randomized clinical trials have shown lower rates of treatment failure (defined as worsening tachycardia, tachypnea, or oxygen requirement) for children with bronchiolitis treated with HFNC compared with low-flow oxygen therapy.^19,20^ These trials did not show reduction in the rates of PICU transfer or intubation and invasive mechanical ventilation (IMV).^19,20^ Importantly, these trials used HFNC predominantly in acute care settings rather than exclusively in PICUs. In contrast, some observational studies in North America have shown that HFNC use is associated with increased PICU admissions for bronchiolitis.^7,16,21,22^ One important limitation of most prior database studies has been the inability to clearly classify patients receiving HFNC therapy due to inconsistencies in the *International Statistical Classification of Diseases, Tenth Revision, Procedure Coding System*.^7,16^ This situation has made it impossible to directly examine whether increases in PICU admissions for bronchiolitis are associated with an increase in HFNC use.

Given the increasing HFNC use for bronchiolitis and progressive PICU bed shortages, we sought to characterize changes in the use of HFNC, NIV, and IMV with data obtained from a retrospective multicenter PICU database to examine whether these changes were associated with an increase in PICU admissions. We hypothesized that HFNC use and PICU admissions increased over the past decade without a contemporaneous reduction in the use of IMV. We also sought to identify factors related to HFNC and NIV success and failure.

## Methods

### Study Design and Participants

The Akron Children’s Hospital’s institutional review board deemed this cross-sectional study exempt from review and the informed patient consent requirement due to the use of deidentified data. The study followed the Strengthening the Reporting of Observational Studies in Epidemiology (STROBE) guideline.

We used the Virtual Pediatric Systems (VPS) database, an anonymized, quality-controlled database containing information from approximately 200 PICUs.^23,24^ For the main analysis, we included PICU encounters for children aged younger than 2 years with a primary reason for admission of viral bronchiolitis (VPS star code 466) between January 1, 2013, and December 31, 2022. The star codes are proprietary groupings provided by VPS similar to other diagnosis-related groups. The database began collecting HFNC use data in 2009; however, this was not mandatory until 2017. To avoid overestimating the magnitude of change in the use of HFNC, we included only data from 27 PICUs that collected HFNC data for all years between 2013 and 2022, as some units may have used HFNC before 2017 but did not report it to VPS.

Encounter-level variables were extracted from the VPS database for the eligible population and included admission year and quarter, age category (birth to ≤28 days or 29 days to <2 years), weight, sex, pathogen (RSV, other virus, or unknown), duration of symptoms prior to PICU admission, source of PICU admission (emergency department or other), history of cardiac disease, PICU and hospital length of stay (LOS), time-stamped information on respiratory support (HFNC, IMV, or NIV) and extracorporeal membrane oxygenation (ECMO) received, Pediatric Risk of Mortality III (PRISM III) score (range, 0-71, with higher scores indicating a higher probability of in-hospital mortality),^25^ and mortality. Race and ethnicity were not examined, as they are of limited biologic value in critical bronchiolitis, and prior work has shown discordance between administrative databases and self-reported race and ethnicity.^26,27,28,29^

### Statistical Analysis

Encounters were described with summary statistics. Continuous variables were compared with Wilcoxon rank sum test or Student *t* test as appropriate. Categorical variables were compared with χ^2^ tests as appropriate. Encounters were grouped based on the type of respiratory support received. Linear regression was used to analyze the association between admission year and absolute numbers of encounters stratified by the maximum level of respiratory support required (in increasing order, HFNC, NIV, IMV, or ECMO).

For outcomes assessment, HFNC was defined as successful when patients were weaned to low-flow oxygen therapy or room air and did not require escalation to NIV or IMV or restarting HFNC within 48 hours of stopping HFNC use. To avoid classification errors due to variable documentation, patients needed to have at least 4 hours recorded as off therapy to be considered stopped and restarted. As NIV is generally considered a “higher level” of support than HFNC, NIV was defined as successful when patients were weaned from NIV to HFNC, low-flow oxygen therapy, or room air and did not require restart of NIV or IMV within 48 hours of stopping NIV use. To avoid patient-level interdependence, only the success of the first episode of NIV or HFNC use per encounter was considered. For both analyses, success was based on the maximum level of respiratory support required within 48 hours of stopping previous respiratory support. Very few patients received negative pressure ventilation, so data for this therapy were combined with those for positive pressure NIV. Time receiving HFNC or NIV therapy was plotted using age-stratified Kaplan-Meier plots and the difference between rates of failure (defined as not meeting the criteria for success) was determined by the log-rank test. Multivariable logistic regression using automated stepwise variable selection to minimize the Akaike information criterion was performed to analyze factors associated with HFNC or NIV success.^30^ Models were trained on repeated k-fold cross-validation (10 folds, 3 repeats). For both HFNC and NIV failure, age and weight were collinear, but weight had greater explanatory power (as it was available as a continuous rather than categorical variable); therefore, age was eliminated in stepwise selection. No other variables were removed. All statistical analysis was performed in R, version 4.3.2 (R Project for Statistical Computing). A *P* < .05 was set for statistical significance in all comparisons.

We conducted 2 sensitivity analyses. First, because PICU admissions for respiratory viruses have increased in toddlers and young children after the COVID-19 pandemic,^16^ we included patients aged 0 through 5 years admitted to the PICU with bronchiolitis during the study period. Second, because the main analysis may underestimate the magnitude of change in HFNC use by including only PICUs with data for HFNC use over the entire study period (some PICUs may have not used HFNC in 2013 and begun between 2013 and 2022), we included all PICUs with admissions for bronchiolitis between 2013 and 2022.

## Results

### Patient Demographics

Included in the analysis were 33 816 encounters for patients (13 628 females [40.3%] and 20 186 males [59.7%]) with bronchiolitis between January 1, 2013, and December 31, 2022, across 27 PICUs. Only 1910 children (5.6%) were aged 28 days or younger, with the remaining 31 906 children (94.4%) aged 29 days to less than 2 years. A total of 7615 of 15 518 patients (49.1%) had RSV infection and 1522 of 33 816 (4.5%) had preexisting cardiac disease. Median (IQR) weight was 7.5 (5.1-9.8) kg. The median (IQR) PICU and hospital LOS were 2.2 (1.3-3.9) days and 4.1 (2.7-6.9) days, respectively. In all, 33 757 children (99.8%) survived to hospital discharge. Full demographic and missing data are shown in Table 1.

**Table 1.** Patient Demographics

Characteristic	No./total No. (%)
Age	
Birth to ≤28 d	1910/33 816 (5.6)
29 d to <2 y	31 906/33 816 (94.4)
Sex	
Female	13 628/33 816 (40.3)
Male	20 186/33 816 (59.7)
Unknown	2/33 816 (<0)
Weight, median (IQR), kg	7.5 (5.1-9.8)
Cardiac disease	1522/33 816 (4.5)
Infection	
Pathogen	
RSV	7615/33 816 (22.5)
Other virus	7903/33 816 (23.4)
Unknown	18 298/33 816 (54.1)
Admission source	
Emergency department	22 758/33 816 (67.3)
Other^a^	11 058/33 816 (32.7)
Duration of symptoms prior to PICU admission, median (IQR), d	0.9 (0.6-1.7)
Unknown	22 479/33 816 (66.5)
Hospitalization outcomes	
Received HFNC	28 141/33 816 (83.2)
Received NIV	10 466/33 816 (30.9)
Received IMV	4446/33 816 (13.1)
Received ECMO	74/33 816 (0.2)
PICU length of stay, median (IQR), d	2.2 (1.3-3.9)
Hospital length of stay, median (IQR), d	4.1 (2.7-6.9)
Unknown	3454/33 816 (10.2)
Survival to hospital discharge (%)	33 757/33 816 (99.8)

Abbreviations: ECMO, extracorporeal membrane oxygenation; HFNC, high-flow nasal cannula; IMV, invasive mechanical ventilation; NIV, noninvasive ventilation; PICU, pediatric intensive care unit; RSV, respiratory syncytial virus.

^a^ Includes direct admissions to the PICU, interhospital transfers, and intrahospital transfers (eg, from the ward).

### Trends in PICU Admissions and Severity of Illness

Admissions to the PICU increased from 1706 in 2013 to 5204 in 2022 (350 admissions per year [95% CI, 170-531 admissions per year]; *P* = .005). Median (IQR) PICU LOS decreased from 2.6 (1.3 to 4.9) days in 2013 to 2.0 (1.3-3.4) days in 2022 (−0.15 days [95% CI, −0.17 to −0.13 days]; *P* < .001). A total of 32 994 encounters (97.6%) had PRISM III data available. There was a small, but statistically significant, decrease in the PRISM III score over time (median [IQR] PRISM III score, 0 [0-3] in 2013 vs 0 [0-1] in 2022; *P* < .001, Wilcoxon rank sum test).

### Trends in Maximum Level of Respiratory Support Required

**Figure 1.**
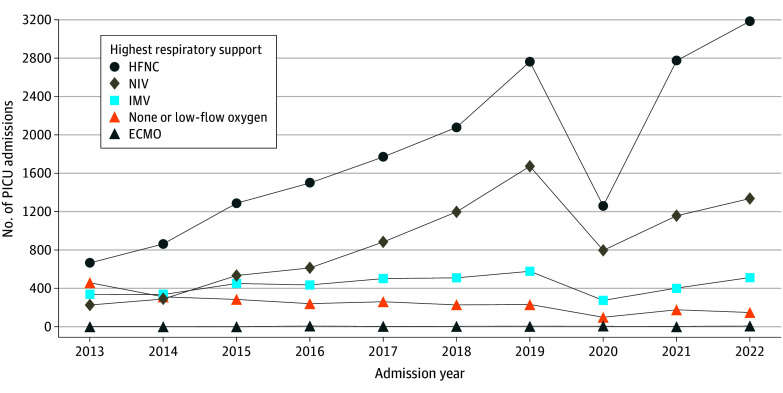
Bronchiolitis Pediatric Intensive Care Unit (PICU) Admissions Over Time Stratified by Maximum Level of Respiratory Support Required ECMO indicates extracorporeal membrane oxygenation; HFNC, high-flow nasal cannula; IMV, invasive mechanical ventilation; and NIV, noninvasive ventilation.

The number of admissions grouped by maximum level of respiratory support required is shown in Figure 1. Encounters with HFNC as the maximum level of respiratory support required increased from 668 in 2013 to 3186 in 2022 (242 [95% CI, 139-345] encounters per year; *P* = .002). Encounters with NIV as the maximum level of respiratory support required increased from 230 in 2013 to 1339 in 2022 (126 [95% CI, 64-189] encounters per year; *P* = .004). Encounters with IMV as the maximum level of respiratory support required did not significantly change (from 341 in 2013 to 516 in 2022; 10 [95% CI, −11 to 31] encounters per year; *P* = .39). The number of encounters requiring ECMO doubled from 5 to 11, but this change was not statistically significant (1 [95% CI, 0-1] encounter per year; *P* = .05).

### HFNC Outcomes

**Figure 2.**
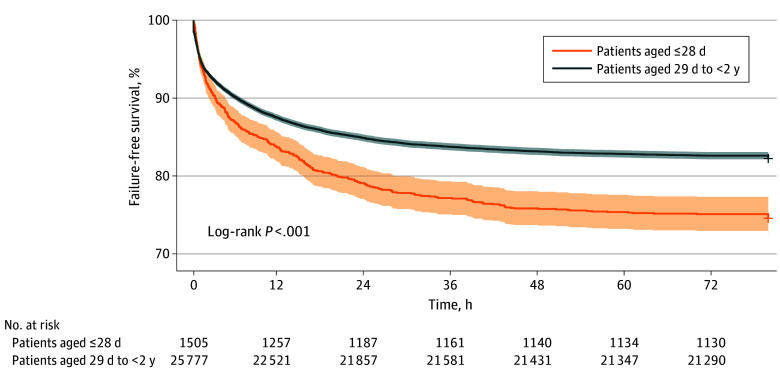
Kaplan-Meier Analysis of High-Flow Nasal Cannula Therapy for Bronchiolitis Stratified by Age Failure was defined as restarting high-flow nasal cannula therapy after weaning or escalation to noninvasive ventilation, invasive mechanical ventilation, or extracorporeal membrane oxygenation.

Time-stamped respiratory support data for 27 282 of 28 141 HFNC encounters (96.9%) were available for outcome analysis. Overall, 22 318 patients (81.8%) were successfully weaned from HFNC to low-flow oxygen therapy or room air, 431 (1.6%) were restarted on HFNC, 3057 (11.2%) were escalated to NIV, and 1476 (5.4%) were escalated to IMV or ECMO (4964 [18.2%] total failures to any modality). Success with HFNC increased from 820 of 1027 encounters (79.8%) in 2013 to 3693 of 4399 encounters (84.0%) in 2022 (*P* = .002). The median (IQR) duration of HFNC therapy was 30.2 (17.8-48.4) hours for patients who were weaned. The median (IQR) duration of HFNC ranged from 20.7 (11.2-34.0) hours to 58.5 (40.6-80.0) hours across the 27 PICUs included (a 2.8-fold variability). The median (IQR) time to failure of HFNC therapy was 4.4 (0.8-14.5) hours. Results of Kaplan-Meier analysis are shown in Figure 2 and eFigure 1 in Supplement 1. Children aged 28 days or younger had a greater probability of HFNC failure than children aged 29 days to less than 2 years (log-rank *P* < .001). Lower weight, cardiac disease, higher PRISM III score, and PICU admission from outside the emergency department were associated with greater odds of HFNC failure in multivariable regression analyses (Table 2; eTable 1 in Supplement 1). Receiving HFNC as step-down therapy after NIV or IMV was associated with lower odds of HFNC failure (Table 2; eTable 1 in Supplement 1).

**Table 2.** Multivariable Logistic Regression Analysis for Factors Associated With HFNC or NIV Failure

Characteristic	HFNC failure		NIV failure	
	OR (95% CI)	*P* value	OR (95% CI)	*P* value
Weight, kg	0.87 (0.86-0.89)	<.001	0.90 (0.87-0.92)	<.001
Cardiac disease				
No	1 [Reference]	NA	1 [Reference]	NA
Yes	1.53 (1.26-1.86)	<.001	1.43 (1.05-1.95)	.02
Admission source				
Emergency department	1 [Reference]	NA	1 [Reference]	NA
Other than emergency department^a^	1.77 (1.61-1.94)	<.001	1.27 (1.08-1.47)	.003
Received NIV, IMV, or ECMO before HFNC				
No	1 [Reference]	NA	1 [Reference]	NA
Yes	0.28 (0.24-0.32)	<.001	NA	NA
Higher PRISM III score	1.21 (1.18- 1.23)	<.001	1.24 (1.20-1.27)	<.001
Received HFNC before NIV				
No	1 [Reference]	NA	1 [Reference]	NA
Yes	NA	NA	1.20 (1.02-1.41)	.02
Received IMV or ECMO before NIV				
No	1 [Reference]	NA	1 [Reference]	NA
Yes	NA	NA	0.74 (0.52-1.03)	.08

Abbreviations: ECMO, extracorporeal membrane oxygenation; HFNC, high-flow nasal cannula; IMV, invasive mechanical ventilation; NA, not applicable; NIV, noninvasive ventilation; OR, odds ratio; PRISM, Pediatric Risk of Mortality.

^a^ Includes direct admissions to pediatric intensive care unit, interhospital transfers, and intrahospital transfers (eg, from the ward).

### NIV Outcomes

**Figure 3.**
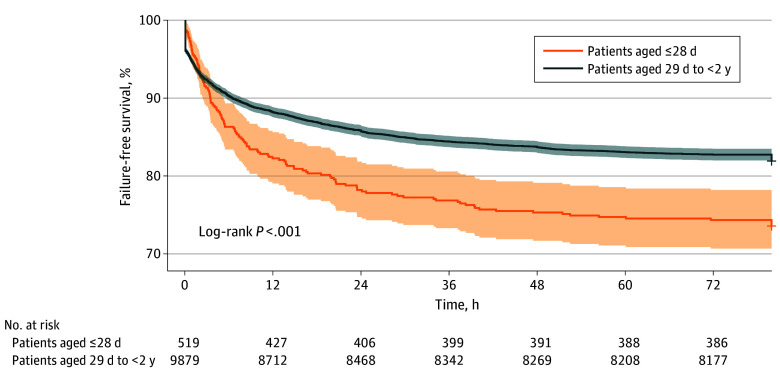
Kaplan-Meier Analysis of Noninvasive Ventilation Therapy for Bronchiolitis Stratified by Age Failure was defined as restarting noninvasive ventilation therapy after weaning or escalation to invasive mechanical ventilation or extracorporeal membrane oxygenation.

Time-stamped respiratory support data were available for outcome analysis for 10 398 of 10 466 NIV encounters (99.4%). Overall, 8476 patients (81.5%) were successfully weaned from NIV, 787 (7.6%) were restarted on NIV, and 1135 (10.9%) were escalated to IMV or ECMO (1922 [18.5%] total failures to any modality). Success with NIV increased from 224 of 306 encounters (73.2%) in 2013 to 1335 of 1589 encounters (84.0%) in 2022 (*P* < .001). The median (IQR) duration of NIV was 32.0 (16.8-55.0) hours for patients who were weaned. The median (IQR) duration of NIV ranged from 14.1 (4.1-37.7) hours to 71.7 (46.1-117.2) hours across the 27 PICUs included (a 5.1-fold variability). The median (IQR) time to failure of NIV was 4.9 (0.6-19.9) hours. Results of the Kaplan-Meier analysis are shown in Figure 3 and eFigure 2 in Supplement 1. Children aged 28 days or younger had a greater probability of NIV failure than children aged 29 days to less than 2 years (log-rank *P* < .001). Lower weight, cardiac disease, higher PRISM III score, PICU admission from outside the emergency department, and receiving HFNC before NIV were associated with greater odds of HFNC failure in multivariable regression analyses (Table 2; eTable 2 in Supplement 1). Receiving NIV as step-down therapy after IMV was associated with lower odds of NIV failure (Table 2; eTable 2 in Supplement 1).

### Sensitivity Analyses

The sensitivity analysis of patients aged 0 through 5 years included 37 568 encounters. Demographics are shown in eTable 3 in Supplement 1. Total PICU admissions increased from 1875 to 5959 (399 [95% CI, 199-600] encounters per year; *P* = .004). PICU admissions for children aged 2 through 5 years increased from 169 of 1875 (9.0%) to 755 of 5959 (12.7%) (*P* < .001). The numbers of encounters according to the maximum level of respiratory support required are shown in eFigure 3 in Supplement 1. Encounters requiring HFNC increased from 717 in 2013 to 3684 in 2022 (281 [95% CI, 164-398] encounters per year; *P* = .002). Encounters requiring NIV increased from 253 in 2013 to 1418 in 2022 (136 [95% CI, 71-201] encounters per year; *P* = .003). Encounters requiring IMV did not significantly change from 383 in 2013 to 589 in 2022 (12 [95% CI, −11 to 36] encounters per year; *P* = .34). Children aged 2 through 5 years had lower rates of HFNC and NIV failure than those aged 28 days or younger or those aged 29 days to less than 2 years (eFigures 4 and 5 in Supplement 1).

The sensitivity analysis for all admissions to PICUs for bronchiolitis included 94 061 encounters across 163 PICUs. Demographics are shown in eTable 4 in Supplement 1. Total admissions increased from 4495 to 15 195 (925 [95% CI, 369-1480] encounters per year; *P* = .01). The number of encounters according to the maximum level of respiratory support required are shown in eFigure 6 in Supplement 1. Encounters requiring HFNC increased 5.9-fold from 1439 in 2013 to 8512 in 2022 (709 [95% CI, 396-1023] encounters per year; *P* = .002). Encounters requiring NIV increased from 461 in 2013 to 2802 in 2022 (372 [95% CI, 210-534] encounters per year; *P* = .002). Encounters requiring IMV did not significantly change from 894 in 2013 to 1671 in 2022 (37 [95% CI, −39 to 113] encounters per year; *P* = .37).

## Discussion

This retrospective cross-sectional analysis of 27 PICUs found that the number of PICU admissions for bronchiolitis in children aged younger than 2 years increased 3-fold between 2013 and 2022. This increase was associated with a 4.8-fold increase in HFNC use and a 5.8-fold increase in NIV use as the maximum level of respiratory support required, with no significant change in IMV use. Reasons for these trends are likely multifactorial. Epidemiologic data show that the population-based hospitalization rate for bronchiolitis increased between 2018 and 2024.^31^ Simultaneously, centralization of pediatric care resulted in more children being transferred from community facilities to children’s hospitals and a decrease in locally available pediatric hospital beds.^32,33,34,35,36^ Among children’s hospitals, prior work has shown an increasing proportion of PICU admissions for all conditions, especially for bronchiolitis.^6,7,16^ Although influenced by atypical seasonality during the COVID-19 pandemic, PICU admissions for bronchiolitis have continued to rise since 2021.^16^ Findings of previous studies^6,7,16,31,32,33,34,35,36^ combined with those of the present study suggest that increases in HFNC and NIV use are likely due to multiple factors, including a rising population-based PICU admission rate, increased consolidation of pediatric admissions to children’s hospitals, a rising proportion of children with bronchiolitis being admitted to the PICU, and an increase in the proportion of patients admitted to the PICU receiving noninvasive respiratory support. Thus, solutions aimed at reducing PICU burden need to consider multiple components in the chain of care.

Whether increased HFNC use contributes to increased PICU admissions remains unclear. Two randomized clinical trials of HFNC therapy predominantly used HFNC for treating bronchiolitis in acute care wards rather than PICUs.^19,20^ With this strategy, HFNC use may lower PICU admission rates. However, other trials of HFNC therapy in bronchiolitis found nonsignificantly higher PICU transfer rates despite improved vital signs in patients treated with HFNC compared with standard oxygen therapy.^19,20^ Additionally, clinical HFNC use has been variable, with many hospitals restricting its use to PICUs due to resource allocation or the ability to rapidly respond when the patient’s condition deteriorates.^37,38^ This restriction may contribute to PICU volume. An interrupted time series analysis from Canada suggested that HFNC use was associated with an increase in PICU admission rates.^22^ Whether increased HFNC use is associated with increased or decreased PICU use may depend on implementation strategy. Thus, we advocate for further study on risk stratification of patients receiving HFNC to potentially reduce PICU resource strain rather than deimplementation of HFNC.

As in prior studies,^7,22^ increased PICU use in the present study was not associated with worsening severity of illness over time as judged by PRISM III scores or hospital or PICU LOS. Because bronchiolitis often results in isolated respiratory failure rather than multiorgan dysfunction, validated PICU illness severity scores, such as PRISM III, may not adequately capture the range of bronchiolitis severity.^39^ Although a bronchiolitis-specific score is under development, external validation is needed before it can be routinely incorporated into database studies and clinical effectiveness research.^39^ Despite imperfect severity of illness adjustment, it seems biologically implausible that bronchiolitis severity has increased linearly for the past decade. Thus, taken together with findings from prior studies,^7,22^ findings of the current study suggest that changes in clinical practices are at least partially responsible for the striking increase in HFNC and NIV use over the past decade.

The failure rate for HFNC therapy was 18% in the present study compared with 12%^20^ and 14%^19^ in prior randomized clinical trials for HFNC. Our higher failure rate may reflect a higher proportion of children with chronic diseases. While children with cardiac disease were not excluded from prior randomized clinical trials of HFNC therapy for bronchiolitis,^19,20^ the incidence of cardiac disease in those trials was approximately 2%^20^ compared with 4.5% in the present study.

Another important finding of the present study was a 5.8-fold increase in NIV use. While prior work has shown increases in NIV use over the past decade, those studies were unable to reliably differentiate HFNC from NIV.^6,7,16^ The present study confirms that both HFNC and NIV use are rising without reductions in IMV use. While previous trials have compared HFNC and NIV use, the present study is the first multicenter analysis to evaluate risk factors for NIV failure in bronchiolitis.^40,41^ Similar to prior single-center work,^42,43^ we found higher severity of illness was associated with higher odds of HFNC and NIV failure. Characterizing which patients are likely to be successfully treated with NIV remains an important goal of PICU precision medicine.

Patients who were successfully weaned from HFNC or NIV had median durations of therapy of 30.2 and 32.0 hours, respectively. The 2 randomized clinical trials of HFNC use in bronchiolitis had mean durations of 20 hours and 43 hours.^19,20^ Given that both of these trials had similar HFNC weaning protocols, the differences in these durations may reflect either differences in patient selection or clinician tolerance of subjective respiratory distress while weaning from HFNC. The median duration of therapy ranged from 20.7 to 58.5 hours across the 27 PICUs included in the present study, suggesting that local practices might affect the duration of HFNC use. A positive deviance quality improvement approach may identify high-performing hospitals and determine whether the duration of HFNC use may be safely shortened.^44,45^

Consistent with prior research, we found that the percentage of children aged 2 through 5 years admitted to the PICU with bronchiolitis increased in our sensitivity analysis.^16^ These children appear to have a milder illness than those aged younger than 2 years, with a lower incidence of HFNC and NIV failure. However, the COVID-19 pandemic was associated with an increase in LRTI admissions in children aged 2 through 5 years, perhaps because they were not exposed during infancy.^16^ Thus, while US Food and Drug Administration approval of monoclonal antibodies against RSV and maternal vaccination are likely to decrease future bronchiolitis admissions in children, future studies should also examine LRTI admission rates in this population as a potential unintended consequence of monoclonal antibody use.^46^

### Limitations

This study has limitations. First, because the VPS database did not include HFNC as a mandatory data field until 2017, the main analysis was limited to 27 PICUs that contributed HFNC use data annually between 2013 and 2022. This limitation may overestimate the use of HFNC before 2017. A sensitivity analysis including all PICUs with data on bronchiolitis showed a 5.9-fold increase in HFNC use during the study period compared with a 4.8-fold increase found in the main analysis. Second, this database only captures patients admitted to PICUs. As HFNC is used in the ward settings in some hospitals, this study may underestimate HFNC use. Third, determining severity of illness in retrospective database studies is challenging, particularly for a low-mortality illness, such as bronchiolitis. While PRISM III scores decreased over time with increasing PICU admissions for bronchiolitis, it is possible that patients’ degree of respiratory distress increased in a way that was not captured in the database. Fourth, the VPS database predominantly includes PICUs from the US; thus, trends described here may not be generalizable to other countries.

## Conclusions

In this retrospective cross-sectional study of data obtained from the VPS database, admission volume for bronchiolitis in patients aged younger than 2 years increased 3-fold between 2013 and 2022 across 27 PICUs. During this time, there was a 4.8-fold increase in HFNC use and a 5.8-fold increase in NIV use, without a concomitant decrease in IMV use. Increased use of NIV and HFNC may be contributing to increased PICU admissions without reducing the need for IMV. There was 2.8-fold and 5.1-fold variability in the duration of HFNC and NIV support, respectively, provided across hospitals. Further research is needed to standardize approaches to HFNC and NIV support in bronchiolitis to reduce resource strain.

## Notes

Supplement 1. **eTable 1.** Demographics Stratified by High-Flow Nasal Cannula Outcome **eTable 2.** Demographics Stratified by Noninvasive Ventilation Outcome **eTable 3.** Demographics of Sensitivity Analysis Including Patients <6 Years Old **eTable 4.** Demographics of Sensitivity Analysis Including all Pediatric Intensive Care Units **eFigure 1.** Cumulative Incidence of High-Flow Nasal Cannula Weaning or Failure Stratified by Age **eFigure 2.** Cumulative Incidence of Noninvasive Ventilation Weaning or Failure Stratified by Age **eFigure 3.** Pediatric Intensive Care Unit Admissions for Bronchiolitis Over Time Stratified by Maximum Level of Respiratory Support Required for Sensitivity Analysis Including Patients <6 Years Old **eFigure 4.** Kaplan-Meier Analysis of High-Flow Nasal Cannula Therapy for Bronchiolitis Stratified by Age for Sensitivity Analysis Including Patients <6 Years Old **eFigure 5.** Kaplan-Meier Analysis of Noninvasive Ventilation for Bronchiolitis Stratified by Age for Sensitivity Analysis Including Patients <6 Years Old **eFigure 6.** Pediatric Intensive Care Unit (PICU) Admissions for Bronchiolitis Over Time Stratified by Maximum Level of Respiratory Support Required for Sensitivity Analysis Including All PICUs

Supplement 2.
Data Sharing Statement
