# Supplementary material for: Respiratory Support Practices for Bronchiolitis in the Pediatric Intensive Care Unit
Source: JAMA Netw Open. 2024 May 10;7(5):e2410746. doi: 10.1001/jamanetworkopen.2024.10746 (PMC11087830; doi:10.1001/jamanetworkopen.2024.10746)

Supplemental Online Content

Pelletier JH, Maholtz D, Hanson C, et al. Respiratory support practices for bronchiolitis in the pediatric intensive care unit. *JAMA Netw Open*. 2024;7(5):e2410746. doi:10.1001/jamanetworkopen.2024.10746

eTable 1. Demographics Stratified by High-Flow Nasal Cannula Outcome

eTable 2. Demographics Stratified by Noninvasive Ventilation Outcome

eTable 3. Demographics of Sensitivity Analysis Including Patients <6 Years Old

eTable 4. Demographics of Sensitivity Analysis Including all Pediatric Intensive Care Units

eFigure 1. Cumulative Incidence of High-Flow Nasal Cannula Weaning or Failure Stratified by Age

eFigure 2. Cumulative Incidence of Noninvasive Ventilation Weaning or Failure Stratified by Age

eFigure 3. Pediatric Intensive Care Unit Admissions for Bronchiolitis Over Time Stratified by Maximum Level of Respiratory Support Required for Sensitivity Analysis Including Patients <6 Years Old

eFigure 4. Kaplan-Meier Analysis of High-Flow Nasal Cannula Therapy for Bronchiolitis Stratified by Age for Sensitivity Analysis Including Patients <6 Years Old

eFigure 5. Kaplan-Meier Analysis of Noninvasive Ventilation for Bronchiolitis Stratified by Age for Sensitivity Analysis Including Patients <6 Years Old

eFigure 6. Pediatric Intensive Care Unit (PICU) Admissions for Bronchiolitis Over Time Stratified by Maximum Level of Respiratory Support Required for Sensitivity Analysis Including All PICUs

This supplemental material has been provided by the authors to give readers additional information about their work.

eTable 1. Demographics Stratified by High-Flow Nasal Cannula Outcome

		HFNC Outcome		
**Characteristic**	**Overall**, N = 27,282[1]	**Successfully Weaned**, N = 22,318[1]	**Failed**, N = 4,964[1]	**p-value**
**Age Category**				<0.001[2]
Neonate Birth to 29 days	1,505 (5.5%)	1,122 (5.0%)	383 (7.7%)	
Infant 29 days to < 2 years	25,777 (94.5%)	21,196 (95.0%)	4,581 (92.3%)	
**Sex**				0.005[2]
Female	10,930 (40.1%)	8,854 (39.7%)	2,076 (41.8%)	
Male	16,351 (59.9%)	13,463 (60.3%)	2,888 (58.2%)	
(Unknown)	1	1	0	
**Weight (Kilograms)**	7.4 (5.2, 9.7)	7.7 (5.4, 10.0)	6.1 (4.5, 8.5)	<0.001[3]
**Virus**				<0.001[2]
RSV	6,840 (52.6%)	5,574 (51.1%)	1,266 (60.2%)	
Other Virus	6,169 (47.4%)	5,332 (48.9%)	837 (39.8%)	
(Unknown)	14,273	11,412	2,861	
**Admission Source**				<0.001[2]
Admitted via Emergency Department	18,006 (66.0%)	15,406 (69.0%)	2,600 (52.4%)	
Other Admission	9,276 (34.0%)	6,912 (31.0%)	2,364 (47.6%)	

Characteristic	**Overall**, N = 27,282[1]	HFNC Outcome<br>**Successfully Weaned**, N = 22,318[1]	 **Failed**, N = 4,964[1]	p-value
**Cardiac Disease**	1,097 (4.0%)	770 (3.5%)	327 (6.6%)	<0.001[2]
**Respiratory Support Before HFNC**	5,219 (19.1%)	4,730 (21.2%)	489 (9.9%)	<0.001[2]

[1] n (%); Median (IQR)

[2] Pearson's Chi-squared test

[3] Wilcoxon rank sum test

© 2024 Pelletier JH et al. *JAMA Network Open.*

eTable 2. Demographics Stratified by Noninvasive Ventilation Outcome

Characteristic	Overall, N = 10,398[1]	NIV Outcome<br>Successfully Weaned, N = 8,476[1]	 Failed, N = 1,922[1]	p-value
**Age Category**				<0.001[2]
Neonate Birth to 29 days	519 (5.0%)	382 (4.5%)	137 (7.1%)	
Infant 29 days to < 2 years	9,879 (95.0%)	8,094 (95.5%)	1,785 (92.9%)	
**Sex**				0.2[2]
Female	4,246 (40.8%)	3,435 (40.5%)	811 (42.2%)	
Male	6,152 (59.2%)	5,041 (59.5%)	1,111 (57.8%)	
**Weight (Kilograms)**	7.5 (5.2, 9.8)	7.8 (5.4, 10.0)	6.2 (4.4, 8.9)	<0.001[3]
**Virus**				<0.001[2]
RSV	2,163 (43.0%)	1,731 (40.4%)	432 (58.2%)	
Other Virus	2,862 (57.0%)	2,552 (59.6%)	310 (41.8%)	
(Unknown)	5,373	4,193	1,180	
**Admission Source**				<0.001[2]
Admitted via Emergency Department	6,612 (63.6%)	5,496 (64.8%)	1,116 (58.1%)	
Other Admission	3,786 (36.4%)	2,980 (35.2%)	806 (41.9%)	
**Cardiac Disease**	464 (4.5%)	324 (3.8%)	140 (7.3%)	<0.001[2]

Characteristic	**Overall**, N = 10,398[1]	NIV Outcome<br>**Successfully Weaned**, N = 8,476[1]	**Failed**, N = 1,922[1]	p-value
**IMV Before NIV**	476 (4.6%)	354 (4.2%)	122 (6.3%)	<0.001[2]
**HFNC Before NIV**	3,545 (34.1%)	2,755 (32.5%)	790 (41.1%)	<0.001[2]

[1] n (%); Median (IQR)

[2] Pearson's Chi-squared test

[3] Wilcoxon rank sum test

eTable 3. Demographics of Sensitivity Analysis Including Patients <6 Years Old

Characteristic	N = 37,568[1]
**Age Category**	
Neonate Birth to 29 days	1,910 (5.1%)
Infant 29 days to < 2 years	31,906 (84.9%)
Child 2 years to < 6 years	3,752 (10.0%)
**Sex**	
Female	15,378 (40.9%)
Male	22,188 (59.1%)
(Unknown)	2
**Race**	
American Indian or Alaska Native	464 (1.3%)
Asian American or Pacific Islander	1,282 (3.6%)
Hispanic	4,843 (13.7%)
Non-Hispanic Black	7,616 (21.5%)
Non-Hispanic White	18,276 (51.6%)
Other	2,944 (8.3%)
(Unknown)	2,143
**Weight (Kilograms)**	8.0 (5.4, 10.4)
**Pathogen**	
RSV	8,447 (48.6%)
Other Virus	8,931 (51.4%)
(Unknown)	20,190
**Admission Source**	

**Characteristic**	**N = 37,568**[1]
Admitted via Emergency Department	25,495 (67.9%)
Other Admission	12,073 (32.1%)
**Cardiac Disease**	1,778 (4.7%)
**Duration of Symptoms prior to ICU Admission (Days)**	0.9 (0.6, 1.7)
(Unknown)	25,101
**ICU Length of Stay (Days)**	2.2 (1.3, 3.9)
**Hospital Length of Stay (Days)**	4.1 (2.7, 6.9)
(Unknown)	3,833
**Survival to Discharge**	37,497 (99.8%)

[1] n (%); Median (IQR)

eTable 4. Demographics of Sensitivity Analysis Including all Pediatric Intensive Care Units

Characteristic	N = 94,061[1]
**Age Category**	
Neonate Birth to 29 days	6,440 (6.8%)
Infant 29 days to < 2 years	87,621 (93.2%)
**Sex**	
Female	38,103 (40.5%)
Male	55,953 (59.5%)
(Unknown)	5
**Race**	
American Indian or Alaska Native	892 (1.1%)
Asian American or Pacific Islander	3,478 (4.2%)
Hispanic	12,066 (14.5%)
Non-Hispanic Black	16,682 (20.1%)
Non-Hispanic White	42,709 (51.3%)
Other	7,363 (8.9%)
(Unknown)	10,871
**Weight (Kilograms)**	7.1 (4.9, 9.5)
**Pathogen**	
RSV	23,399 (56.2%)
Other Virus	18,201 (43.8%)
(Unknown)	52,461
**Admission Source**	
Admitted via Emergency Department	60,293 (64.1%)

Characteristic	N = 94,061[1]
Other Admission	33,768 (35.9%)
**Cardiac Disease**	4,352 (4.6%)
**Duration of Symptoms prior to ICU Admission (Days)**	1.0 (0.6, 2.1)
(Unknown)	56,743
**ICU Length of Stay (Days)**	2.3 (1.4, 4.1)
**Hospital Length of Stay (Days)**	4.4 (2.8, 7.5)
(Unknown)	9,056
**Survival to Discharge**	93,903 (99.8%)

[1] n (%); Median (IQR)

eFigure 1. Cumulative Incidence of High-Flow Nasal Cannula Weaning or Failure Stratified by Age. The x-axis shows the time on high flow nasal cannula therapy in hours. The y-axis shows the cumulative probability of successful weaning or failure at any given time.

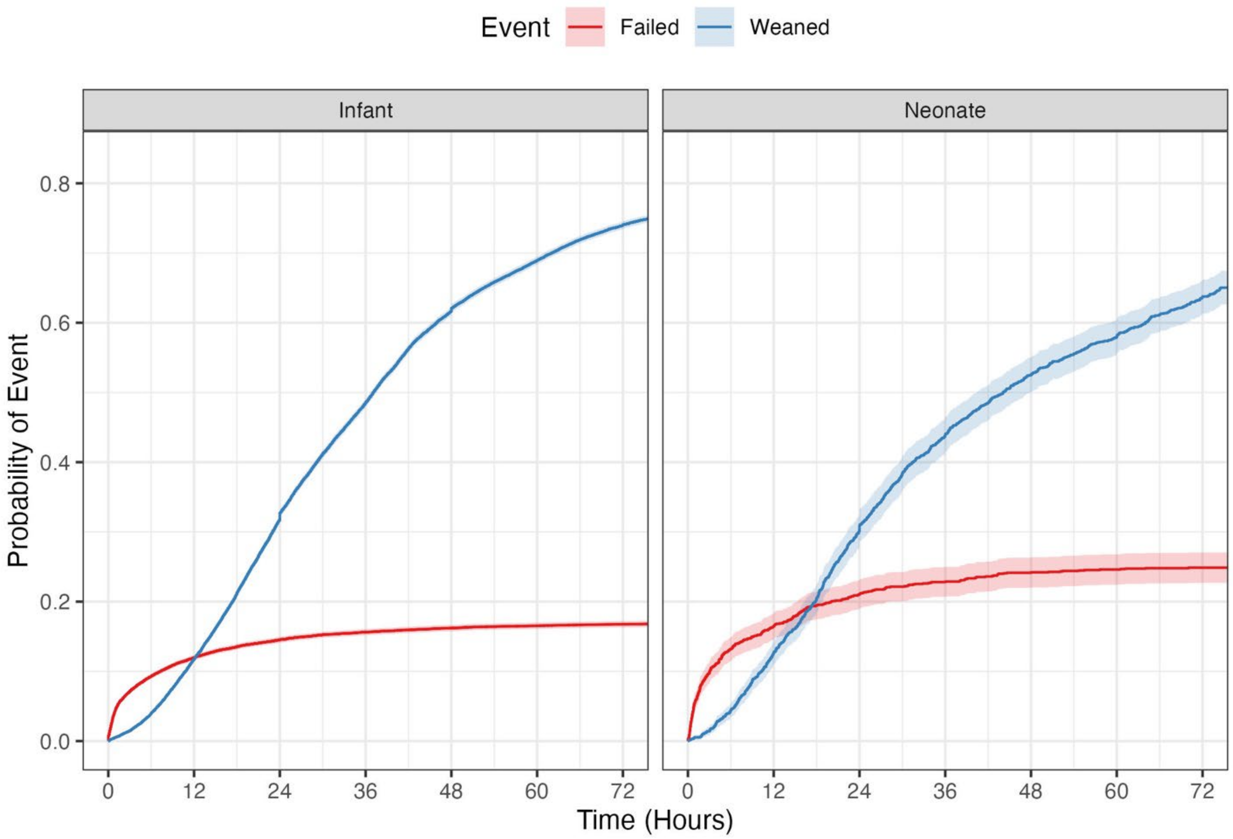

eFigure 2. Cumulative Incidence of Noninvasive Ventilation Weaning or Failure Stratified by Age. The x-axis shows the time on non-invasive ventilation in hours. The y-axis shows the cumulative probability of successful weaning or failure at any given time.

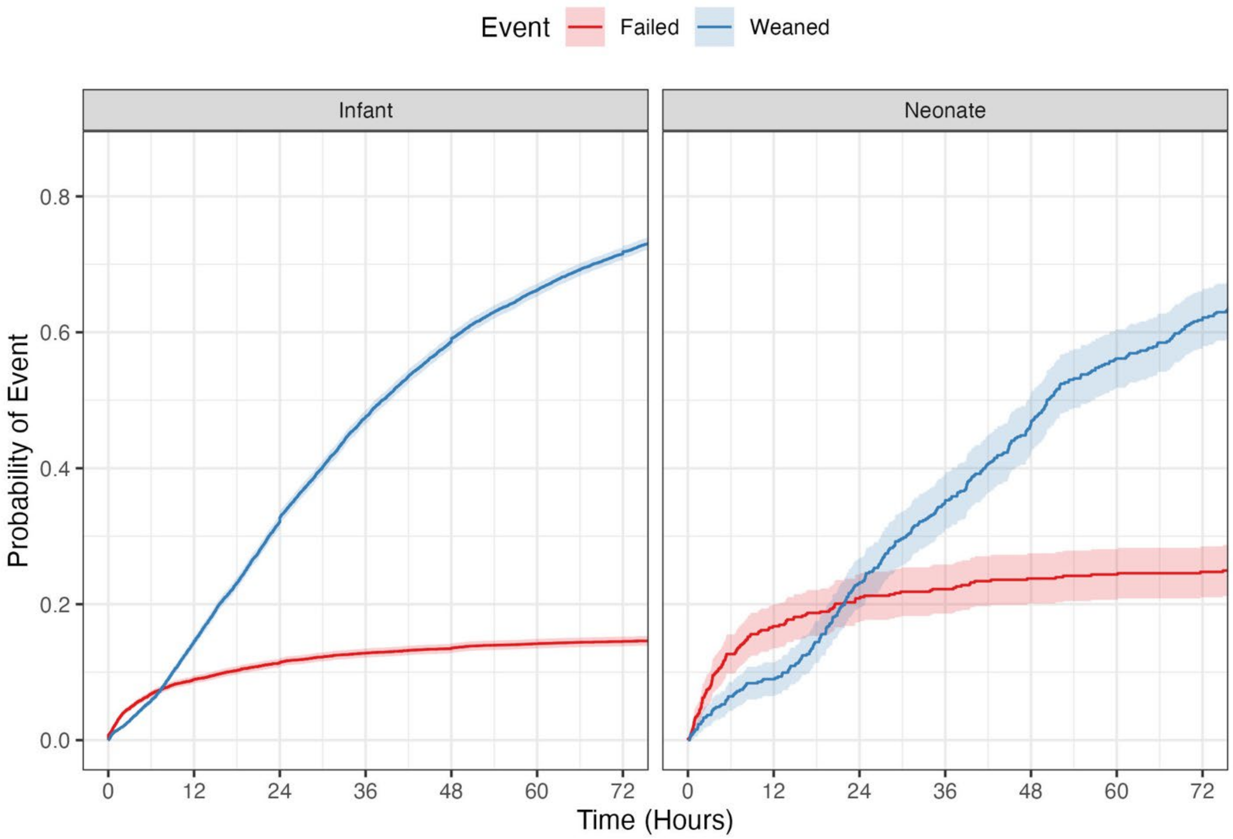

eFigure 3. Pediatric Intensive Care Unit Admissions for Bronchiolitis Over Time Stratified by Maximum Level of Respiratory Support Required for Sensitivity Analysis Including Patients <6 Years Old. Admission year is shown in the x-axis versus the number of admissions on the y-axis. Colors represent the highest level of respiratory support during an admission. HFNC: High-flow Nasal Cannula. NIV: Non-invasive Ventilation. IMV: Invasive Mechanical Ventilation. ECMO: Extracorporeal Membrane Oxygenation.

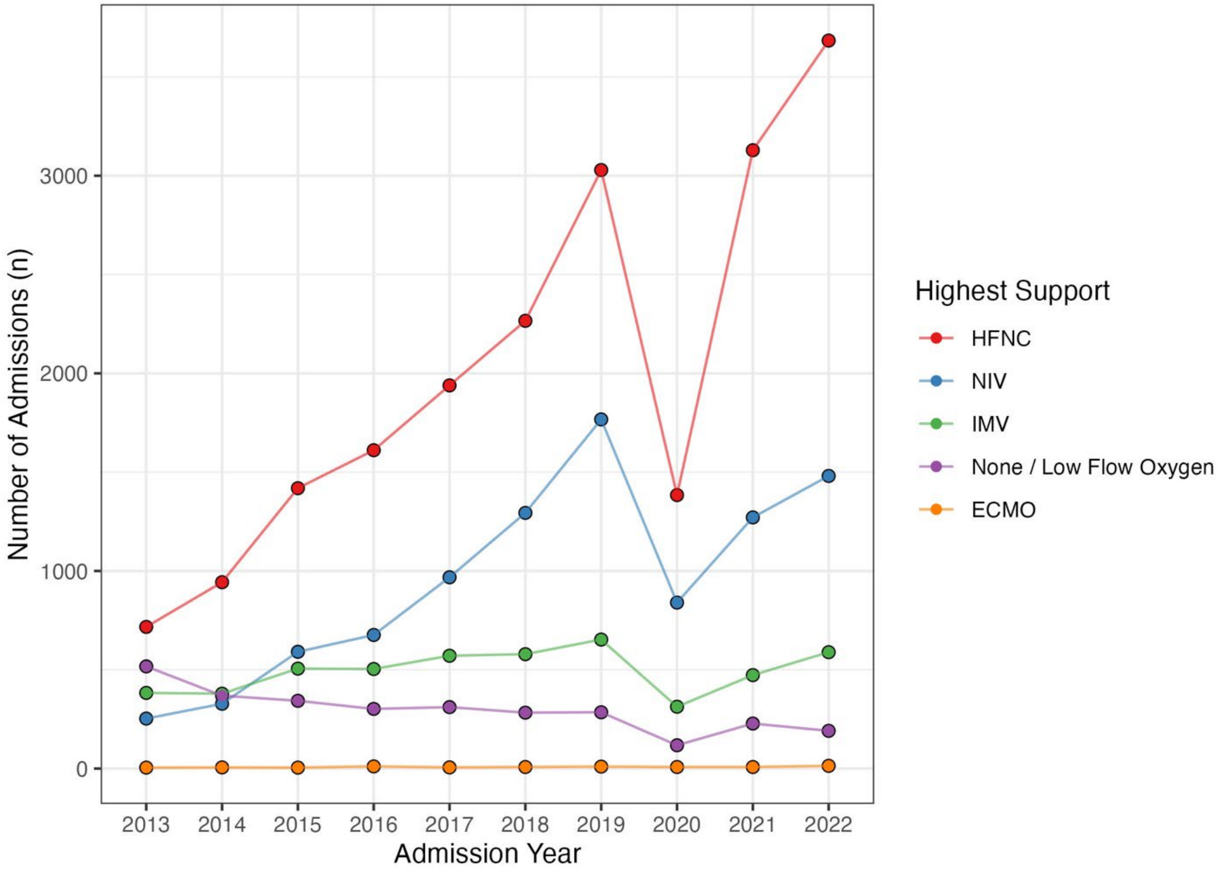

eFigure 4. Kaplan-Meier Analysis of High-Flow Nasal Cannula Therapy for Bronchiolitis Stratified by Age for Sensitivity Analysis Including Patients <6 Years Old. Time is shown on the x-axis versus failure-free survival on the y-axis. Failure was defined as restarting high-flow nasal cannula therapy after weaning, escalation to non-invasive ventilation, escalation to invasive mechanical ventilation, or escalation to extracorporeal membrane oxygenation. The p-value is determined by the log-rank test. The colors show the age groups, where neonate includes patients < 28 days old, infant includes patients 28 days to < 2 years old, and child includes patients 2-5 years old. The table below the x-axis shows the number (%) of patients at risk in each group.

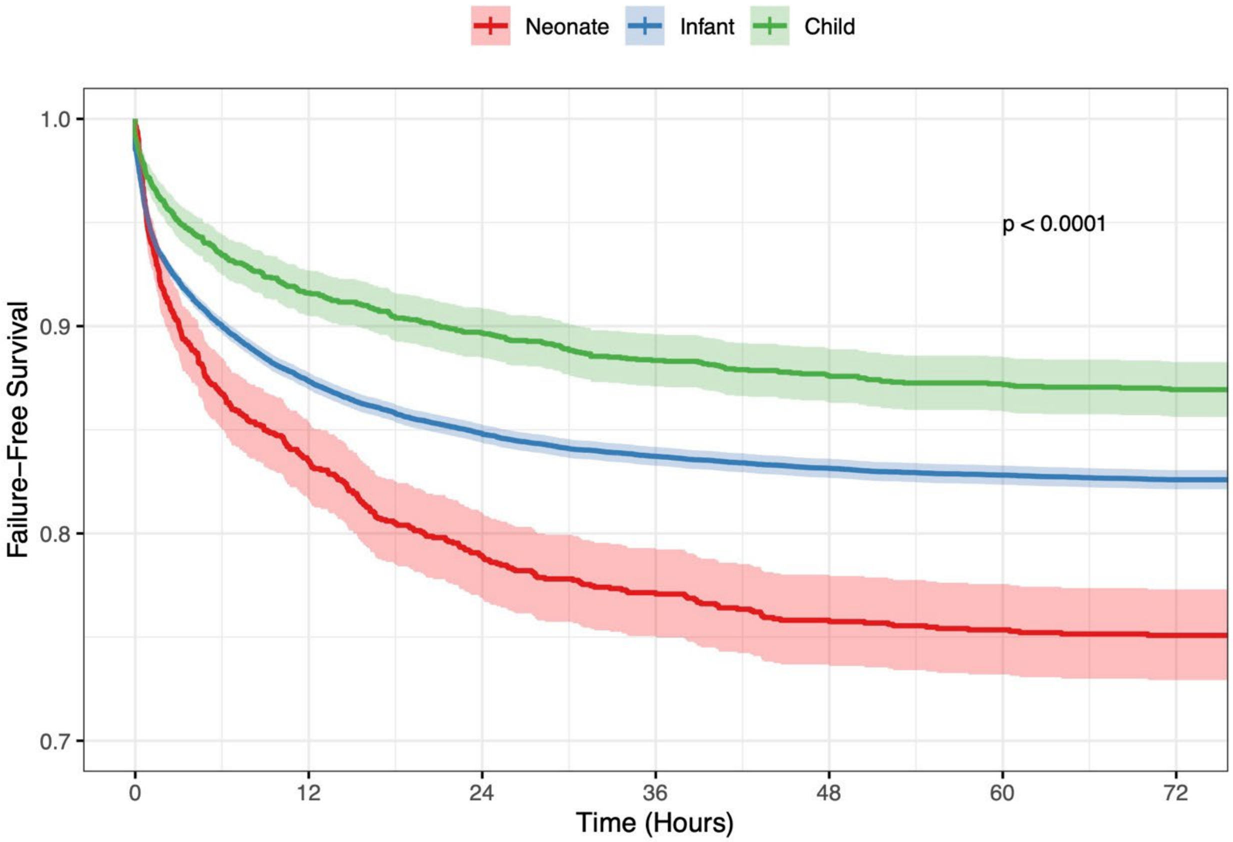

	0	12	24	36	48	60	72
Neonate	1505 (100)	1257 (84)	1187 (79)	1161 (77)	1140 (76)	1134 (75)	1130 (75)
Infant	25777 (100)	22521 (87)	21857 (85)	21581 (84)	21431 (83)	21347 (83)	21290 (83)
Child	2488 (100)	2279 (92)	2231 (90)	2198 (88)	2179 (88)	2170 (87)	2163 (87)

Time (Hours)

eFigure 5. Kaplan-Meier Analysis of Noninvasive Ventilation for Bronchiolitis Stratified by Age for Sensitivity Analysis Including Patients <6 Years Old. Time is shown on the x-axis versus failure-free survival on the y-axis. Failure was defined as restarting high-flow nasal cannula therapy after weaning, escalation to non-invasive ventilation, escalation to invasive mechanical ventilation, or escalation to extracorporeal membrane oxygenation. The p-value is determined by the log-rank test. The colors show the age groups, where neonate includes patients < 28 days old, infant includes patients 28 days to < 2 years old, and child includes patients 2-5 years old. The table below the x-axis shows the number (%) of patients at risk in each group.

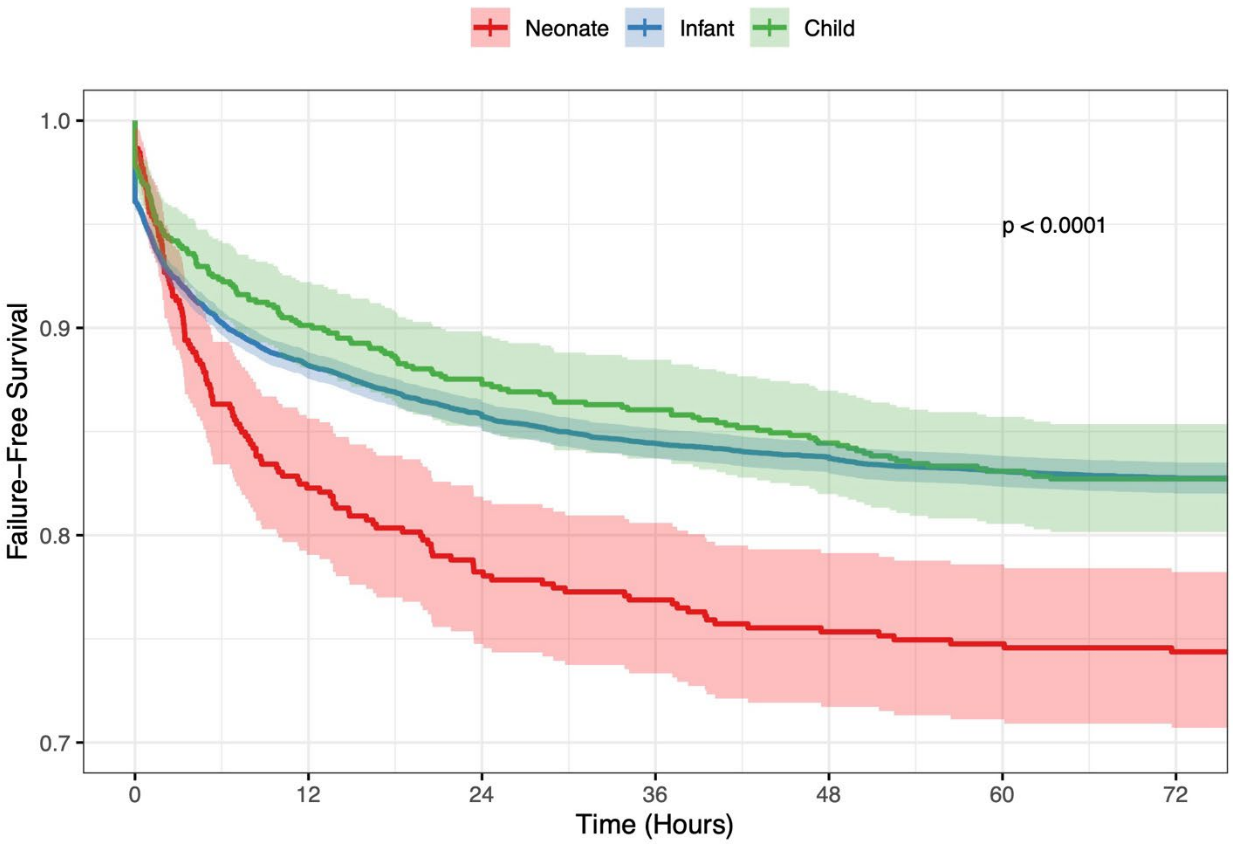

Group	0	12	24	36	48	60	72
Neonate (red)	519 (100)	427 (82)	406 (78)	399 (77)	391 (75)	388 (75)	386 (74)
Infant (blue)	9879 (100)	8712 (88)	8468 (86)	8342 (84)	8269 (84)	8208 (83)	8177 (83)
Child (green)	810 (100)	730 (90)	707 (87)	697 (86)	684 (84)	673 (83)	670 (83)

Time (Hours)

eFigure 6. Pediatric Intensive Care Unit (PICU) Admissions for Bronchiolitis Over Time Stratified by Maximum Level of Respiratory Support Required for Sensitivity Analysis Including All PICUs. Admission year is shown in the x-axis versus the number of admissions on the y-axis. Colors represent the highest level of respiratory support during an admission. HFNC: High-flow Nasal Cannula. NIV: Non-invasive Ventilation. IMV: Invasive Mechanical Ventilation. ECMO: Extracorporeal Membrane Oxygenation.

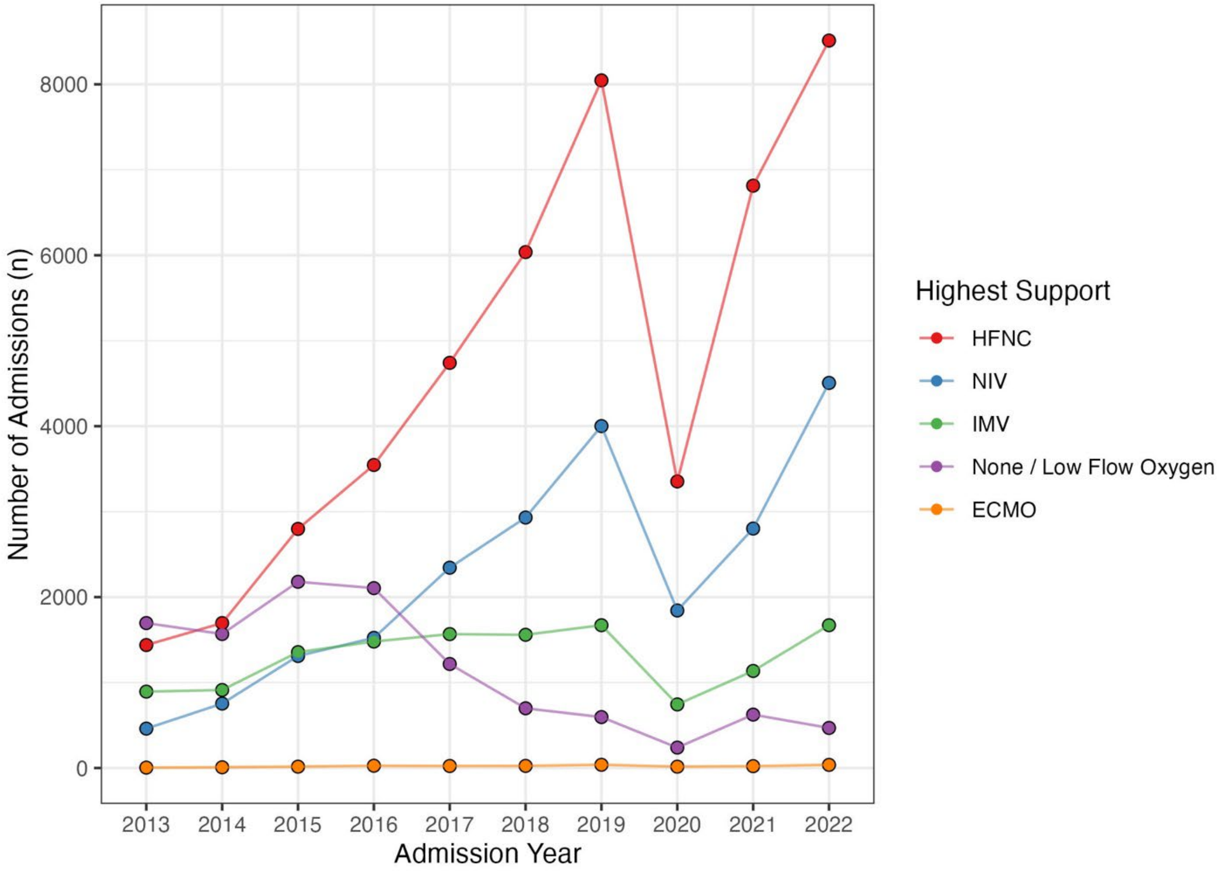

Supplement: Supplement 1. — eTable 1. Demographics Stratified by High-Flow Nasal Cannula Outcome eTable 2. Demographics Stratified by Noninvasive Ventilation Outcome eTable 3. Demographics of Sensitivity Analysis Including Patients <6 Years Old eTable 4. Demographics of Sensitivity Analysis Including all Pediatric Intensive Care Units eFigure 1. Cumulative Incidence of High-Flow Nasal Cannula Weaning or Failure Stratified by Age eFigure 2. Cumulative Incidence of Noninvasive Ventilation Weaning or Failure Stratified by Age eFigure 3. Pediatric Intensive Care Unit Admissions for Bronchiolitis Over Time Stratified by Maximum Level of Respiratory Support Required for Sensitivity Analysis Including Patients <6 Years Old eFigure 4. Kaplan-Meier Analysis of High-Flow Nasal Cannula Therapy for Bronchiolitis Stratified by Age for Sensitivity Analysis Including Patients <6 Years Old eFigure 5. Kaplan-Meier Analysis of Noninvasive Ventilation for Bronchiolitis Stratified by Age for Sensitivity Analysis Including Patients <6 Years Old eFigure 6. Pediatric Intensive Care Unit (PICU) Admissions for Bronchiolitis Over Time Stratified by Maximum Level of Respiratory Support Required for Sensitivity Analysis Including All PICUs [file jamanetwopen-e2410746-s001.pdf]
