# Supplementary material for: Respiratory Support Practices for Bronchiolitis in the Pediatric Intensive Care Unit
Source: JAMA Netw Open. 2024 May 10;7(5):e2410746. doi: 10.1001/jamanetworkopen.2024.10746 (PMC11087830; doi:10.1001/jamanetworkopen.2024.10746)
Supplement: Supplement 2. — Data Sharing Statement [file jamanetwopen-e2410746-s002.pdf]

Data Sharing Statement

Pelletier. Respiratory Support Practices for Bronchiolitis in the Pediatric Intensive Care Unit. *JAMA Netw Open*. Published May 10, 2024. doi:10.1001/jamanetworkopen.2024.10746

Data
Data available: No

Additional Information
Explanation for why data not available: The data used in this article cannot be shared directly by the authors as it is owned by Virtual Pediatric Systems (VPS). Hospitals interested in utilizing VPS data can find more information at https://myvps.org/join-vps/
